# Effects of Combination Treatments with Astaxanthin-Loaded Microparticles and Pentoxifylline on Intracellular ROS and Radiosensitivity of J774A.1 Macrophages

**DOI:** 10.3390/molecules26175152

**Published:** 2021-08-25

**Authors:** Eleonora Binatti, Gianni Zoccatelli, Francesca Zanoni, Giulia Donà, Federica Mainente, Roberto Chignola

**Affiliations:** 1 Department of Biotechnology, University of Verona, Strada Le Grazie 15-CV1, I-37134 Verona, Italy; gianni.zoccatelli@univr.it (G.Z.); federica.mainente@univr.it (F.M.); roberto.chignola@univr.it (R.C.); 2 Sphera Encapsulation S.r.l., Strada Le Grazie 15-CV1, I-37134 Verona, Italy; zanoni@spheraencapsulation.com (F.Z.); lab@spheraencapsulation.com (G.D.)

**Keywords:** pentoxifylline, astaxanthin, particle encapsulation, oxidative stress, ionizing radiations, macrophages, radiation-induced fibrosis

## Abstract

Radiation-induced fibrosis (RIF) is a serious, yet incurable, complication of external beam radiation therapy for the treatment of cancer. Macrophages are key cellular actors in RIF because of their ability to produce reactive oxidants, such as reactive oxygen species (ROS) and inflammatory cytokines that, in turn, are the drivers of pro-fibrotic pathways. In a previous work, we showed that phagocytosis could be exploited to deliver the potent natural antioxidant astaxanthin specifically to macrophages. For this purpose, astaxanthin encapsulated into µm-sized protein particles could specifically target macrophages that can uptake the particles by phagocytosis. In these cells, astaxanthin microparticles significantly reduced intracellular ROS levels and the secretion of bioactive TGFβ and increased cell survival after radiation treatments. Here we show that pentoxifylline, a drug currently used for the treatment of muscle pain resulting from peripheral artery disease, amplifies the effects of astaxanthin microparticles on J774A.1 macrophages. Combination treatments with pentoxifylline and encapsulated astaxanthin might reduce the risk of RIF in cancer patients.

## 1. Introduction

Radiation-induced fibrosis (RIF) is the most common long-term complication in cancer radiotherapy, and it affects a significant proportion of the ~60% cancer patients that receive radiation as part of their therapeutic regimens [1]. The onset of RIF occurs between 1–4 months and 1–2 years after radiotherapy and it progresses in severity with time [2]. RIF does not regress spontaneously, and to date, there are no effective treatments for RIF prevention and/or treatment [3]. Investigations with patients and experimental animals have demonstrated a significant reduction of RIF with antioxidant treatments administered after radiation [4]. The pathogenesis of RIF is still not fully understood, although it is known that phagocytic cells, such as macrophages, play a key role in RIF for their ability to produce oxidant chemical species, in particular reactive oxygen species (ROS), and inflammatory molecules that in turn trigger the fibrotic pathways in injured tissues [5].

Pentoxifylline (3,7-dimethyl-1-(5-oxohexyl)purine-2,6-dione, PTX) is a derivative of xanthine. PTX was approved by FDA in 1984 for the treatment of muscle pain resulting from peripheral artery disease, but it is also used off-label for the treatment of leg ulcers [6]. Indeed, PTX is known to improve the rheological properties of blood and wound healing, and to have anti-inflammatory and antioxidant activity [7].

PTX has also been investigated as a radioprotector in vitro and in vivo because it can increase blood flow and tissue oxygenation upon oral administration [8]. It has provided satisfactory results in terms of RIF resolution but only at high doses (i.e., 800 mg/day over a 6-month treatment period [8]) that are not tolerated by patients and that can cause, among the side effects, serious gastrointestinal disorders [8,9]. Thus PTX alone cannot be used to cure RIF. Vitamin E is a well-known antioxidant, and it can effectively scavenge reactive oxygen species produced by radiation treatments. Moreover, its deficiency is associated with dysfunction of connective tissue repair [9]. When administered orally to patients its effects on RIF were only modest, but given in combination with PTX tablets, significantly reduced fibrosis and related clinical symptoms such as skin tightness, dyspnea, and pain [2].

Astaxanthin (ASX) is a xanthophyll ketocarotenoid with an antioxidant potential 100 times higher than vitamin E [10]. We previously explored in vitro whether ASX could be effective against RIF [11]. ASX was encapsulated into µm-sized protein particles that can only be taken up by cells through phagocytosis, and in this way, we could target them specifically to macrophages [11]. In these cells, ASX microparticles significantly reduced intracellular ROS levels and, importantly, the secretion of bioactive transforming growth factor β (TGFβ) [11]. The role of this pro-inflammatory cytokine is central to fibrosis because it promotes fibroblasts recruitment, and local deposition of extracellular matrix components [12]. In addition, it inhibits the expression of antioxidant enzymes in target cells, further contributing to intracellular ROS accumulation [12].

Given these encouraging results and the outcomes of combination treatments with PTX and vitamin E discussed above, we explored whether PTX could also enhance the effects of ASX when given in combination to macrophages. We found that PTX could indeed improve the antioxidant and radio-protective activities of ASX microparticles.

## 2. Results

Full details concerning the synthesis of ASX-loaded microparticles along with their complete biochemical and biological characterization have been given in reference [11]. In this work we used the same preparations and in this section we describe new data concerning PTX and its interactions with ASX microparticles.

### 2.1. Cytotoxicity of PTX on J774A.1 Macrophages

**Figure 1:**
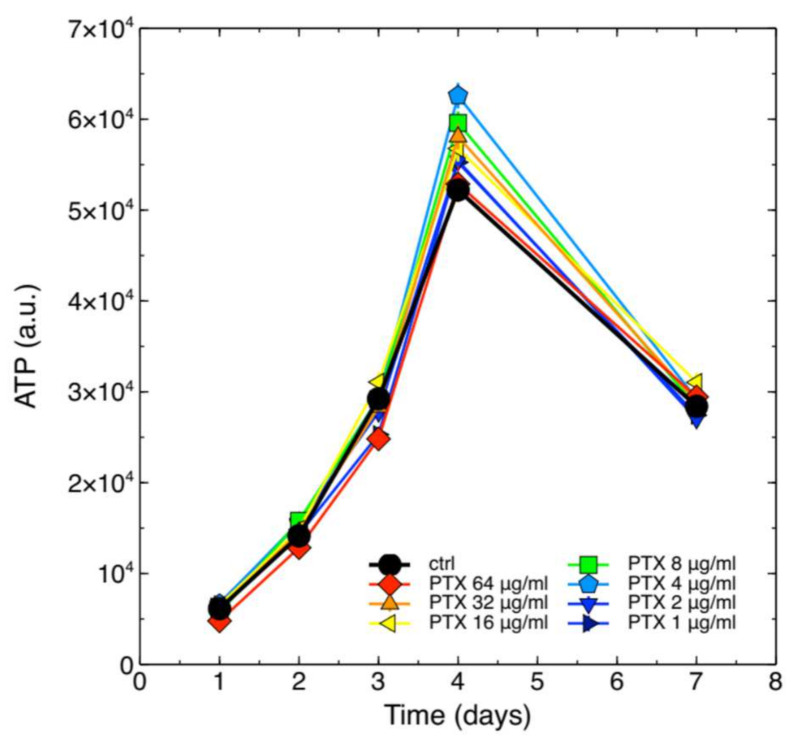
Growth kinetics of J774A.1 cells exposed to different concentrations of PTX. The drug was administered to the cells at the indicated final concentrations at the beginning of the growth assays, and ATP was then measured. Cell populations were also left untreated as controls (black circles marked as ctrl). The error bars correspond to calculated standard errors and are not clearly visible in this figure because they are masked by the symbols (min. and max. coefficient of variation, CV = 100∗SE/mean, 0.75% and 6.0%, respectively).

In our previous work, we showed that ASX microparticles were not cytotoxic for J774A.1 macrophage cells [11]. To the best of our knowledge no information is available on the safety of PTX in this cell system. We therefore assayed whether PTX could alter the growth kinetics of these cells. Intracellular ATP content was measured at different time points of the growth curve obtained with independent cell populations continuously exposed to different PTX concentrations, and the results are shown in Figure 1.

The data show that PTX, at all assayed concentrations, was not cytotoxic for J774A.1 cells.

### 2.2. PTX Does Not Alter Phagocytosis of ASX-Loaded Microparticles

Central to our treatment strategy is the idea that antioxidant molecules can be delivered specifically to macrophages (and other phagocytes) if loaded into particles of appropriate size that can be taken up by these cells through phagocytosis (see also our analyses carried out by confocal microscopy and reported in [11]). PTX has been reported to inhibit the phagocytosis induced by latex particles (~0.8 µm diameter) in monocytes and polymorphonuclear leukocytes in a dose-dependent manner. The critical PTX concentration at which the inhibitory activity became significant was 10 µg/mL [13]. We therefore assayed whether a slightly lower PTX concentration, namely 8 µg/mL, could inhibit phagocytosis of ASX microparticles in J774A.1 cells.

**Figure 2:**
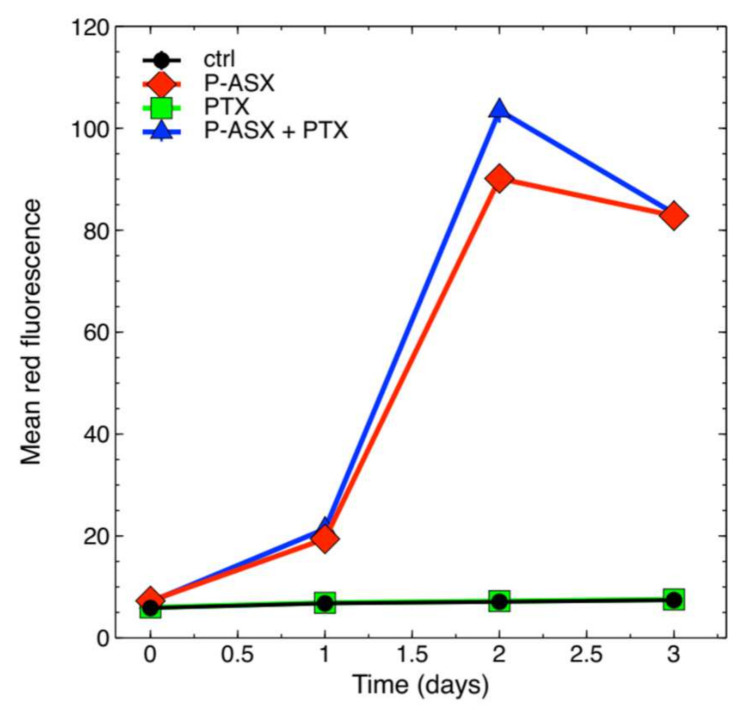
Phagocytosis kinetics of ASX microparticles. The cells were exposed to ASX microparticles (P-ASX) and were either treated or left untreated with PTX at a final concentration of 8 µg/mL. Phagocytosis was measured by flow cytometry thanks to the fluorescence emission of the ASX-containing oleoresin encapsulated into the protein particles. In this figure cell autofluorescence of control untreated cells (ctrl) or of cells treated with PTX only is also shown. Values are given as mean ± SE. The error bars correspond to calculated standard errors and are not clearly visible in this figure because they are masked by the symbols (min. and max. coefficient of variation, CV=100∗SE/mean, 0.45% and 5.1%, respectively).

Figure 2 shows that 8 µg/mL PTX did not inhibit phagocytosis of ASX microparticles in J774A.1 macrophages.

### 2.3. Effects of PTX and ASX Microparticles on Intracellular ROS Levels

**Figure 3:**
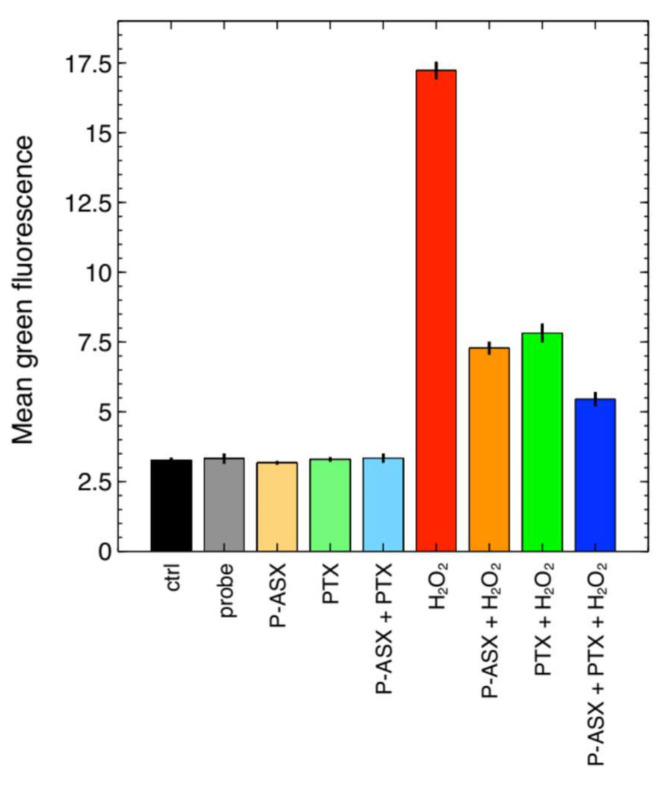
Effects of PTX and ASX-loaded microparticles on intracellular ROS as evaluated by flow cytometry with the DCF-DA probe. Cell fluorescence was almost equal for untreated cells (cell autofluorescence, ctrl) or cells treated with DCF-DA (probe), ASX microparticles (P-ASX), PTX or with P-ASX and PTX. Cell fluorescence significantly increased in cells loaded with DFC-DA and treated with H_2_O_2_, but treatments with PTX and ASX microparticles given alone or in combination significantly suppressed the effects of H_2_O_2_ (see Figure 4 and Table 1 for statistical details). Data are the mean ± SE of 4 independent samples.

PTX has been reported to have antioxidant activity [7], and more importantly to have a synergic effect with other antioxidants, such as vitamin E, in combined treatments [14]. We previously showed that ASX microparticles could significantly reduce intracellular ROS levels in J774A.1 cells treated with H_2_O_2_ to induce oxidative stress [11], and we therefore wondered whether PTX could also improve the effects of ASX particles. Figure 3 shows that the intracellular ROS levels in J774A.1 macrophages decreased when PTX and ASX microparticles were given alone or in combination.

**Figure 4:**
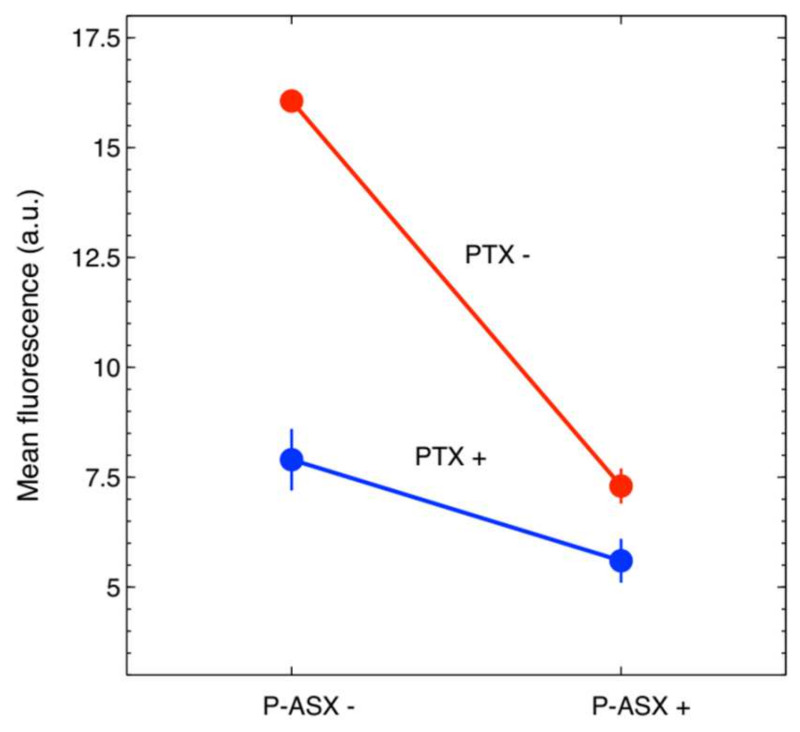
Graphical representation of the synergic effect of PTX and ASX microparticles on intracellular ROS reduction in J774A.1 cells. The two lines would be expected to be parallel if there were no synergic effects [15]. Formally, synergism is demonstrated by two-ways ANOVA statistical analysis (see Table 1).

Two-way ANOVA analyses indicated that PTX and ASX microparticles significantly interacted to reduce the intracellular ROS levels induced by hydrogen peroxide treatment in J774A.1 cells (Figure 4 and Table 1).

### 2.4. Radical-Scavenging Activity of PTX and ASX-Containing Extract in a Cell-Free Assay

Data in Figure 3 and Figure 4 and Table 1 indicate that PTX is an intracellular ROS scavenger. We further investigated its activity in a well-controlled cell-free assay, i.e., ABTS assay, in the attempt to explore whether the drug could react with free radicals on its own. As the control we used ASX-containing extracts that have an acknowledged antioxidant activity [16]. This result is somewhat expected since in a previous study PTX showed poor scavenging activity using DPPH assay [17]. This behavior is probably due to the lack of hydroxyl groups on PTX structure that underpin the scavenging activity of other potent antioxidants, such as vitamin E and phenolic compounds [18].

**Figure 5:**
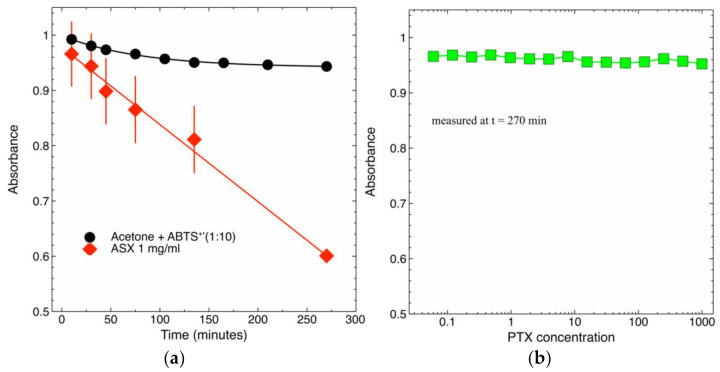
Antioxidant activity of PTX and ASX extracts in a cell free assay. (**a**) Antioxidant kinetics of ASX extracts compared to the spontaneous decay of ABTS cation radicals (ABTS^+^) in acetone solvent. (**b**) Antioxidant kinetics of PTX overlapped those of ABTS^+^^·^ in acetone at all assayed concentrations. For the sake of clarity, in this panel we show only the final time-point, i.e., the measurements carried out after 270 min for all PTX concentrations.

Figure 5 shows that PTX had no radical-scavenging activity at all assayed concentrations.

We therefore concluded that the effects observed for PTX on intracellular ROS levels likely depend on the ability of the drug to activate cellular detoxifying pathways (see also Section 3 for further discussion on this point).

### 2.5. Effects of PTX and ASX Microparticles on Irradiated Cells

Ionizing radiations initiate oxidative stress in biological samples through water radiolysis that ultimately leads to cell death [19]. Since PTX and ASX microparticles cooperated to significantly reduce intracellular ROS (Figure 3 and Figure 4 and Table 1) we asked whether they could also increase the survival of irradiated cell samples when given alone or in combination to cells before radiation treatments.

The clonogenic assay is commonly performed in radiobiology to quantify the radiosensitivity of cell samples. This test is based on the ability of cells to grow in isolation and form colonies [20], but in spite of several attempts we could not obtain well defined colonies with J774A.1 cells (see also reference [11] for further discussion on this point). We therefore resorted to a recently developed method that translates into experiments a probabilistic model of cell survival after radiotherapy and that is based on Poisson statistics [11,21]. The model is described by Equation (1) (see Section 4.8), and the final goal is to estimate the model parameter *S*(*D*) by nonlinear fitting of Equation (1) to experimental data. This parameter defines the survival probability of irradiated clonogenic cells. However, to univocally estimate its values an independent determination of the multiplicative parameter *ε* is required [11,21]. Parameter *ε*, on its turn, defines the clonogenic potential of non-irradiated cells and it can be precisely estimated using limiting dilution assays [11,21].

**Figure 6:**
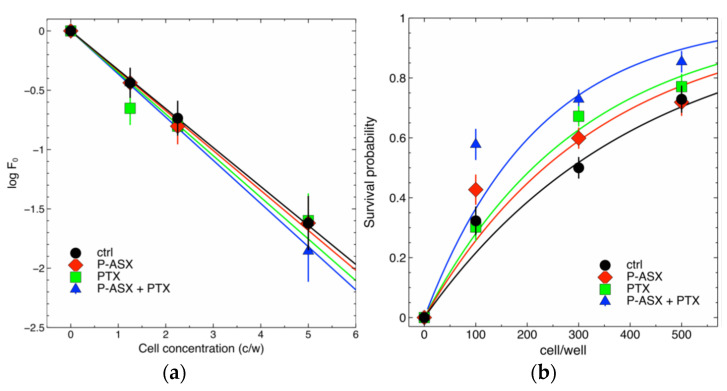
Effects of PTX and ASX microparticles on irradiated J774A.1 macrophages. (**a**) Limiting dilution assays were performed with non-irradiated cells to estimate parameter *ε* in Equation (1) (see the main text for details). Cells were seeded at each indicated density into the wells of 96-well culture plates and treated with ASX microparticles (P-ASX) and PTX, either alone or in combination (P-ASX + PTX), or left untreated (ctrl). *F*_0_ is the fraction of cells where no proliferation was observed at the end of the observation period (~30 days). When the cells are randomly and independently distributed, *F*_0_ is expected to obey Poisson statistics with parameter *ε*. Linear fitting of log-transformed data provided the following estimates: *ε*_ctrl_ = 0.328 ± 0.0325, *ε*_P-ASX_ = 0.336 ± 0.036, *ε*_PTX_ = 0.351 ± 0.036, *ε*_P-ASX+PTX_ = 0.364 ± 0.038. The differences in the estimated parameter values were not statistically significant (ANOVA test, *p* = 0.9). (**b**) Survival probability of independent cell populations treated with ASX microparticles (P-ASX), PTX, P-ASX in combination with PTX (P-ASX + PTX) or left untreated (ctrl) and then irradiated with a dose of 4 Gy γ-rays. Nonlinear fits with Equation (1) allowed to estimate the fraction of the cells surviving radiation treatments (see also the main text for details). The results are: *S*(*D*)_ctrl_ = 0.0071 ± 0.0005 (χ^2^/df = 1.5), *S*(*D*)_P-ASX_ = 0.0086 ± 0.0006 (χ^2^/df = 1.2), *S*(*D*)_PTX_ = 0.0095 ± 0.0007 (χ^2^/df = 1.7), *S*(*D*)_P-ASX+PTX_ = 0.0131 ± 0.001 (χ^2^/df = 2.1). All observed differences in estimated parameter values were statistically significant as evaluated by ANOVA (*p* = 1.5 × 10^−12^) followed by Tukey post-hoc test.

Figure 6 shows the results of our experiments with J774A.1 macrophages. ASX microparticles and PTX, given alone or in combination, did not alter significantly the clonogenic potential of the J774A.1 cells (parameter *ε*) if compared to that of control untreated cells, but significantly increase the survival of irradiated cells (parameter *S*(*D*)). The survival of irradiated cells increased progressively when the cells were treated with ASX microparticles, PTX or ASX particles in combination with PTX.

## 3. Discussion

Oxidative stress is involved in several diseases such as cancer, Alzheimer’s disease, Parkinson’s disease, atherosclerosis, heart failure, fibrosis and, in particular, RIF. It is a systemic pathologic condition caused by altered ROS accumulation in cells and tissues and hence by imbalance between ROS production and elimination through the activation of specific detoxifying mechanisms [22]. Ionizing radiations produce high amounts of ROS in both intracellular and extracellular compartments through water radiolysis [19]. This causes DNA lesions and other life-threatening damages not only to cancerous cells but also to irradiated and non-irradiated normal cells. It is nowadays accepted that radiation generates “danger” signals that propagate from irradiated to non-irradiated cells (the so-called off-target effects). Such molecular signals include ROS, other reactive molecular species, cytokines, ATP and extracellular DNA [23]. These signals, in turn, activate immune cells that, similar to macrophages, can further contribute to ROS production and can initiate and sustain the inflammatory cascade [23]. If uncontrolled, inflammation can cause tissue remodeling and dysfunction [24].

For example, the TGFβ cytokine promotes the recruitment of fibroblasts and local deposition of extracellular matrix components and it represents a key molecular actor in RIF [12].

RIF manifests in ~1/4 of all cancer patients that undergo radiotherapy with clinical consequences that impact the quality of their life [1]. Our previous study showed that ASX microparticles could not only reduce intracellular ROS levels in macrophages but also inhibited the secretion of bioactive TGFβ by these cells [11], indicating—although within the limitations of a pre-clinical in vitro study—that the treatment strategy developed to target specifically phagocytic cells could indeed be effective.

The results of the present study show that the effects of ASX microparticles can be further potentiated by combination treatment with PTX. PTX was nottoxic for the cells, did not interfere with phagocytosis of ASX microparticles, but at the same time could significantly reduce the oxidative stress in J774A.1 macrophages. Cell-free assays, however, clearly showed that PTX had no direct antioxidant activity on its own. It has been reported that PTX may have indirect antioxidant effects in neutrophils where it may reduce the superoxide production via NADPH oxidase [7]. PTX has also been shown to contribute to the maintenance of GSH levels, mitochondrial viability and in general to have protective effects against malathion-induced oxidative damage to rat brain mitochondria in vivo [25]. The antioxidant activity of PTX, therefore, appears to depend on its ability to modulate intracellular detoxifying pathways. Our results are in line with this interpretation of the molecular mechanisms of action of PTX. Importantly, the drug showed synergistic antioxidant effects with ASX microparticles in J774A.1 cells and significantly contributed to increasing the survival of irradiated macrophages. These findings collectively show that both direct and indirect mechanisms can be activated to restore intracellular ROS levels and protect cells from oxidative injury.

PTX has also a well-acknowledged anti-inflammatory activity, since it can inhibit TNFα production and signaling [8]. Given in combination with ASX microparticles, that on their own inhibit active TGFβ release by targeted macrophages, might result in more effective treatments against inflammation and fibrosis. Overall, our previous and present observations justify further investigations with in vitro and in vivo models of fibrosis.

## 4. Materials and Methods

### 4.1. Pentoxifylline and Astaxanthin Loaded Microparticles

Pentoxifylline P1784-100G powder was provided by Sigma-Aldrich (St. Louis, MO, USA). Astaxanthin Oleoresin (ASTAPure^®^ 10% Oleoresin from Haematococcus pluvialis) was provided by Algatech (Ketura, Israel).

The microencapsulation of astaxanthin oleoresin into the whey protein isolate shell was performed by emulsification and solvent evaporation technique described in the PCT/IB2019/05991 application. Chemical and physical properties (e.g., distribution of particles size, storage stability, absorption spectra) of astaxanthin loaded microparticles were given in our previous work [11]. The median particle size was ~2.5 µm.

The concentration of ASX microparticles was expressed as µg of dried powder/mL of cell growth medium. The mass contribution of ASX into the microparticles is approximately 2.9%. Taking into account that the molecular weight of ASX is 596.8 g/mol this corresponds to a concentration of 48.6 nM ASX (µg/mL) dried particles.

When not specified, in all assays the final concentration of PTX was 8 μg/mL (28.7 mM).

### 4.2. Cells and Cell Culture

J774A.1 macrophages were obtained from the European Collection of Authenticated Cell Cultures (ECACC, Salisbury, UK; ECACC number 91051511). Murine macrophages were cultured in RPMI 1640 medium supplemented with foetal bovine serum (FBS), 10 mg/mL gentamicin (Biochrom) and 2 mM glutamine (Sigma-Aldrich, St. Louis, MO, USA) at 37 °C in a humidified 5% CO_2_ atmosphere.

Cell morphology was routinely checked using an Evos (AMG, Life Technology) digital inverted microscope and an Olympus IX51 (Olympus Corporation, Corporate Parkway Center Valley, PA, USA) inverted microscope.

### 4.3. Cytotoxicity Assay

J774A.1 cells were seeded at 5000 cells/well in 96-wells culture plates in 200 µL RPMI medium containing different concentrations of PTX, from 1 µg/mL to 64 µg/mL. At each time point after treatments the intracellular ATP content was quantified using the Cell Titer Glo^®^ Luminescent Cell Viability Assay (Promega, Milan, Italy) following the manufacturer’s specification. Luminescence was measured with an FLX800 Microplate reader (FLX800, Bio-Tek Instruments, Bad Friedrichshall, Germany). All measurements were carried out at least in 4 replicates.

### 4.4. Flow Cytometry

A Guava easyCyte 5 flow cytometer (Merck Millipore, Billerica, MA, USA) was used. The instrument is equipped with a 488 nm, 20 mW, blue laser light. Light scattering is measured by means of a forward scatter (FSC) photodiode and a side scatter (SSC) photomultiplier. Three fluorescence channels, green, yellow, and red, allow to collect cell-associated fluorescence at the same time, thanks to the following filters: green, 525/30 filter; yellow, 583/26; red, 680/30. Instrument calibration was routinely carried out using the Guava EasyCheck kit (Merck Millipore, Billerica, MA, USA) following the manufacturer’s instructions. We routinely collected at least 5000 gated events for analysis.

### 4.5. Phagocytosis Kinetics

J774A.1 cells were seeded at 20,000 cells/well in six-wells plates. Cells were incubated at 37 °C with 56 µg/mL of ASX microparticles for different days, and at each time point, phagocytosis of the microparticles was analyzed by flow cytometry. In fact, ASX oleoresin emits red fluorescence around 600 nm when excited by a blue laser light. In parallel assays, the cells were also treated with 8 µg/mL PTX.

### 4.6. Intracellular ROS Detection

The cell-permeable and H_2_O_2_-sensitive 2′,7′-Dichlorofluorescein diacetate (DCF-DA) fluorescent probe (Sigma-Aldrich, St. Louis, MO, USA) was used to measure intracellular ROS levels. When the diacetate group is cleaved by intracellular esterases the probe is retained into the cells.

Macrophages were seeded in six-well plates at 60,000 cell/wells in 3 mL of growth medium and treated with 56 mg/mL ASX particles, 8 µg/mL PTX or both for 5 h at 37 °C to allow phagocytosis. The cells were then washed twice with PBS 1×, and the medium was replaced with 500 µL of 50 µM DCF-DA. The cells were further incubated for 30 min. After washings with PBS 1× the cells were incubated for 30 min at 37 °C with 3 mL of a solution containing 2.5 mL of growth medium and 0.5 mL of 0.1% *w*/*w* hydrogen peroxide in PBS (0.017% H_2_O_2_ final concentration) to increase intracellular ROS levels and simulate oxidative stress. The fluorescence of the DCF-DA probe was then measured by flow cytometry.

### 4.7. Free Radical Scavenging: ABTS Test

The free radical scavenging capacity of PTX was also studied using the ABTS radical cation de-colorization assay which is based on the reduction of ABTS^+•^ radicals by antioxidants [17].

ABTS (Sigma-Aldrich, St. Louis, MO, USA) was dissolved in PBS to reach a final concentration of 7.4 mM. ABTS radical cation (ABTS^+•^) was produced by a chemical reaction of ABTS with 2.6 mM potassium persulfate (Sigma-Aldrich St. Louis, MO, USA). The reaction was carried out overnight, in the dark, and at room temperature. The ABTS^+•^ solution was then diluted in methanol to reach an absorbance of 0.75 at 734 nm. A BioTek PowerWave HT, microplate spectrophotometer (BioTek Instruments, Inc., Winoosky, VT, USA) was used.

Different concentrations of PTX dissolved in acetone (Sigma-Aldrich St. Louis, MO, USA) (20 µL) were allowed to react with 200 µL of ABTS^+•^ solution into the wells of a 96-well microplate kept in the dark. Absorbance kinetics were measured at room temperature starting at 5 min after initial mixing. All solutions were used the same day of their preparation, and all determinations were carried out in triplicate.

### 4.8. Irradiation of Cell Samples

The cells were seeded at different densities into the wells of 96-well culture plates, 1 plate for each assayed cell density, and treated with 56 µg/mL of ASX microparticles and/or with 8 µg/mL PTX. After 24 h cells were irradiated with a dose of 4 Gy using a Gammacell40 irradiator (Atomic Energy of Canada Limited, Kanata, ON, Canada) equipped with a ^137^Cs source. The dose rate was 0.6654 Gy/min and the measured uniformity was ±1.3% over the entire sample chamber. Both parameters are monitored by the Radiation Protection Service of the University of Verona.

After ~20 days surviving cell populations were determined by careful microscopic inspection and counted. The number of wells containing survivors divided by the total number of seeded wells is an experimental estimate of the overall survival probability *P* of the cells at a given radiation dose, and this probability is expected to vary as the function of cell density *μ* as follows [11,21]:

*P* = 1 − e^−*S*(*D*) *ε μ*^ (1)

where *S*(*D*) is the survival probability of the cells with self-renewing potential (i.e., clonogens) irradiated with a dose *D*, and *ε* is the fraction of clonogens in a population of *μ* cells on average. To quantify the effects of radiation the parameter *S*(*D*) was estimated by nonlinear fitting of experimental data with Equation (1) knowing the fraction of clonogens *ε* in the cell population. This fraction was determined in independent limiting dilution assays as described [11,21].

### 4.9. Statistics

All assays were carried out in 3–4 replicates and repeated at least three times with different cell batches. Data were expressed as mean ± SE, where SE is the standard error of the mean. Statistical analyses and nonlinear regression were carried out using the software Mathematica (v.12, Wolfram Research Inc., Champaign, IL, USA). The reduced χ^2^, i.e., χ^2^/df where df is the number of degrees of freedom, was used to determine the goodness of the nonlinear fits.

## Figures and Tables

**Table 1:** Two-ways ANOVA analysis of the antioxidant effects of PTX and ASX microparticles given alone or in combination to J774A.1 cells.

Treatment	df ^1^	SS	MS	*F*	*p*
P-ASX	1	97.4	97.4	381.9	1.8 × 10^−10^
PTX	1	122.1	122.1	478.9	4.9 × 10^−11^
PTX and ASX	1	41.2	41.2	161.4	2.6 × 10^−8^
Error	12	3.1	0.255		
Total	15	263.8			

^1^ df, degrees of freedom; SS, sum of squares; MS, mean squares.

## Data Availability

Data are contained within the article.

## Sample Availability

Samples of the compounds are available from the authors.
